# Book Reviews

**Published:** 1801-08

**Authors:** 


### CRITICAL RETROSPECT
### OF
### MEDICAL AND PHYSICAL LITERATURE.

---

*Syſtem der Practiſchen Heilkunde*; i. e. Syſtem of Practical Medicine, adapted for Lecturers and for Practical Uſe; by Dr. Chr. W. Hufeland, Profeſſor at Jena, (now Phyſician to the King of Pruſſia at Berlin) Vol. I. General Therapeutic. 1800, pp. 515, in large 8vo. Jena and Leipzic, Fromman.

The celebrated author of the preſent work, on the appearance of which the general attention has been for a long time fixed, intends to unite the various heads of Practical Medicine under the moſt poſſible ſimple points of view, to reduce the phenomena of the diſeaſed ſtate, as well as the remedies and their actions, to the laws of life and organization, as far as we have hitherto penetrated, and and by that means to eftablifh more confiftency in the different parts of medicine and the methods of cure; to remove the contradictory points, to fill the wants and fupply the deficiences in the art of healing by pure experience and unprejudiced obfervation. The ufe of fuch a work is equal to the tafk and the difficulties which attend the execution of it, as not only a philofophic mind and acutenefs of judgment, but an extenfive knowledge of the different medical theories, and obfervation at the bed of the patient, are neceffarily required of him who undertakes fo great and difficult a tafk. It muft be confeffed, that thefe qualities are not united in any man to a higher degree than in Dr. HUFELAND, whofe name fills fuch an honourable place in the annals of medical literature. Although the theories on which the work is grounded may not agree with the ideas of every reader, yet all muft acknowledge the profound practical knowledge and ingenuity of the author, in fupporting his former opinions with ftrong arguments; recommend his love of truth and impartiality in adopting feveral doctrines of the new theory of excitement, of which he, otherwife, profeffes to be an antagonift; in doing juftice to the merits of others, and in expreffing himfelf with modefty and dignity on their refpective theories. The work is likewife written with the fame elegance of ftyle, in which he furpaffes moft of his colleagues, and by which his other works are equally diftinguifhed.

At the end of the Preface, the author adds a fketch of a Bibliotheca Practica, under the following heads. 1. General introduction to practice; here he ranks, firft, "*Frank's epitome de curandis hominum morbis,*" and concludes the lift with *Brown's* elementa medicinæ, and the remark, "caute incede, latet ignes fub cinere dolofo." *Introduction*; fcope of the art of healing, Macrobiotic, Therapeutic in general. *Part* 1. *General Therapeutic.* To cure difeafes is to change the anomalous ftate of an organic body into a normal or found ftate. Ch. i. *Therapeutic of nature.* It muft be allowed, that the organic body poffeffes the faculty of preferving not only itfelf and its life, but of removing any difturbances in the difeafed ftate, and of reftoring the whole to equilibrium; or, in a word, it is in the power of nature to cure difeafes by itfelf without medical affiftance. The vires naturæ medicatrices are proved by the teft of experience and theory, as organifm is endowed with the faculty of felf-prefervation and regeneration. The vires naturæ medicatrices originate properly in the vis vitalis of organic nature, which penetrates, preferves, and vivifies the whole body. In this point of view, a fanative power of nature may indeed be adopted, as the fame conditions which preferve life and its found ftate, to act here for the reftoration of health. It feems, however, that health and the reftoration of health depend on the extraneous impreffions upon the organic body, and on the manner and poffibility of their action on it; on which account phyficians ought not always to rely on the fanative power of nature, and ftill lefs ought it to be adopted as a proper power of organifm.

Dr. HUFELAND propofes, 1. *The laws of incitation,* according ing to which the therapeutic of nature feems to proceed; he farther confiders, 2. *The fympathy of the fingle parts*; 3. *Antagonifmus.* It is a known fact, that a diminifhed or fuppreffed action in any organ is replaced by that of another, and in this way the cure of many difeafes is effected. Thus a fuppreffed febrile action in an intermittent fever may produce exanthemata, diarrhoeas, &c. whereby any harm is prevented, that could refult from that fuppreffion: fuppreffed or retrograde exanthemata often occafion confumption, which is fometimes cured by their being again reftored. Thefe phenomena, however, admit a different explanation, according to the new theory of excitement, as in all the cafes brought forward by Dr. Hufeland in fupport of his opinion, the effential character of the difeafe remains always the fame, degree and form being only changed. In intermittent fevers, for inftance, the effential character is debility; when the degree of debility becomes ftill lefs, another fymptom of afthenic affection fupervenes, as an exanthema; if now the body continues to be debilitated, the eruption perhaps difappears, and a third form of afthenia fucceeds, as dropfy, &c. It cannot, however, hence be concluded, that the cure of the fever is to be afcribed to the eruption, or this to the dropfy, &c. Which way of explaining is more adequate to nature, we leave our readers to judge. 4. *Secretion*, as a principle of the fanative power of nature. 5. *Pathological change of matter.* 6. *Inftinct.* Some objections may be raifed againft adopting this as a principle on which a cure, performed by nature alone, is to be founded; and though there are cafes, where the patients have a particular longing for things that are proper and falutary, or a difguft for fuch things that may do harm, yet it quite as often happens, that they long for things which would injure them. In feveral cafes the averfion for any thing may be as eafily explained by a topical affection in the organs: Thus a patient in a high degree of typhus will have a difguft for all folid food, which he is at this time unable to digeft, the organs of digeftion being difeafed, and not feeling the fenfation of hunger as a natural confequence of their morbid ftate. 7. *Habit and cuftom*, as a principle of the fanative virtue of nature.—Ch. ii. *General theory of cure.* This chapter contains a number of interefting and ingenious ideas, which are at the fame time of practical ufe; but the nature of the whole admits of no extract. Every cure confifts in changing the anomalous ftate of the body into a normal one, or in an alteration that is purpofely produced in the body, for removing the difeafed ftate. The author has adopted feveral ideas of the theory of excitement, but with fome reftrictions, a circumftance which characterifes him as an unprejudiced thinker and a lover of truth.—Ch. iii. *Remedies, their action, and manner of employing them.* After having difcuffed with much ingenuity the manner in which remedies act on matter and on the humours of the body, Dr. Hufeland diftinguifhes the actions of remedies into thofe which act on excitability and into the actions on the organic mixture of matter, that is to fay, remedies act partly by caufing a change in the external condition of life or of the ftimuli,

ſtimuli, partly by cauſing a change in the inner condition of life or in the organization and its mixture, on which the quality and quantity of vital action depend.—What the author ſtates of ſpecific remedies is very true and of much practical uſe. Their action is either confined to a particular organ or proceeds in a particular manner. The ſpecific action, however, does by no means exclude the general action that attends it. — Ch. iv. *Method of cure, and its difference, according to different purposes.* The whole cure ought to be founded on the diagnoſis of diſeaſes as a diſeaſe that we know is eaſily to be cured, though the diagnoſis itſelf is ſubject to many difficulties. Dr. Hufeland propoſes here ſome excellent practical rules concerning the diagnoſtic part of medicine. The ſcope of cure is, 1. To remove the cauſes of diſeaſes, and conſequently to perform a *radical cure.* 2. To remove the effects of the diſeaſe, or the ſymptoms; *palliative cure.* 3. To preſerve the life of the patient, not by removing the diſeaſe itſelf, but by uſing ſuch remedies as have an immediate effect, *cura vitalis;* this cure is employed, 1. When any ſymptom ſupervenes, which threatens a ſudden danger to the operation of life. 2. When the diſeaſe has an advantageous influence for preſerving life. 3. When the diſeaſe itſelf is incurable. The ſcope of medicine is farther, 4. To prevent future diſeaſes, *cura prophylactica,* which is effected by removing a particular diſpoſition of the body to any diſeaſe; for inſtance, a morbid ſenſibility by corroborative medicines, &c.—Ch. v. *Pathogeny and account of the fundamental diſeaſes, with reſpect to therapeutic.* Thoſe diſeaſes deſerve the appellation of fundamental, or primitive diſeaſes, which originate in a change of the operation of life and vital activity, according to the laws of organization. Amongſt the proximate cauſes of them we obſerve, 1. A defective ſtate of the internal conditions of life; i. e. of the chemical mixture, ſtructure, and form of the organic matter; 2. A defective ſtate of the external conditions of life, or of the ſtimuli; 3. A diſturbed equilibrium and harmony in the peculiar actions of the ſingle organs that conſtitute the whole organization. The changes ariſing in the vital activity are either *quantitive,* that is to ſay, with reſpect to the degree, or to ſthenie and aſthenie, or *qualitive;* i. e. with regard to the modality of the effects and the quality of the products. Every organic body enjoys a peculiar organization, to which its mode of acting bears exact proportion, whence, alſo, a peculiarity in its diſeaſed ſtate ariſes.—Ch. vi. *The fundamental methods of medicine.*—Ch. vii. *The exciting method.* To excite is to increaſe the vivacity and ſtrength of the vital exertions. The author here enumerates the proper remedies, and propoſes the beſt mode of applying them.—Ch. viii. *The corroborative method.* To corroborate is to increaſe the quantity of life, or the ſum of internal and external vital exertions, in ſo much that they proceed with a certain degree of force, ſteadineſs, and duration. Some excellent remarks on the uſe of ſtrong acids are added here.—Ch. ix. *The lenient method,* by which we intend to mitigate the too vehement and anomalous vital exertions produced by the diſeaſe.

cafe, or, at leaft, to make them lefs perceptible to the patients: — Ch. x. *The debilitating method.*—Ch. xi. *The fpecific method* relates to a peculiar quality of the difeafed ftate, requiring to be changed by remedies that have a peculiar action upon it. — Ch. xii. *The antagonistic method.* Any affection of a part may be removed by an oppofite affection in another part; this is a law on which that method is founded.—Ch. xiii. *The restorative method,* by means of which fuch fubftances are imparted to organifm as are fit for becoming conftituent parts of the organic mixture.—Ch. xiv. *The evacuant method.*—Ch. xv. *The method for changing and improving the mixture, properties, and proportion of the organic body.*

Such are the contents of the firft volume of this valuable work, which we may rank amongft the beft literary productions on medicine that have lately appeared, and the continuation of which will be ardently expected by the medical world.

---

*Journal de Medicine, Chirurgie, Pharmacie*; i. e. Journal of Medicine, Surgery, and Pharmacy; conducted by the Citizens Corvissart, Le Roux, and Boyer, Profeffor of the School of Medicine. Paris, *Mequignon,* year 9, fmall 8vo.

The phyficians of France have for a long time regretted the difcontinuance of the old *Journal de Medicine,* which was begun in the year 1754, by Vandermond, conducted afterwards by Mr. Le Roux till 1776, continued by Dumanger and Racher to 1781, and by the latter till the year 2 of the republican era, when it ceafed to appear. The wifhes of the public are at laft gratified by the Citizens Corvissart, Le Roux and Boyer, who have begun to publifh the Journal of Medicine we are now announcing, of which a number of about 96 pages is to appear monthly, fix of which will form a volume of about 576 pages. This new Journal of Medicine, which may be confidered as a Continuation of the old Journal de Medicine, is to contain the following heads. 1. Obfervations, memoirs, differtations, &c. on all parts of the art of healing. 2. Extracts, notices, advertifements of all books on the different branches of that fcience, foreign as well as domeftic, which are to appear, or which have been publifhed fince the ceffation of the Journal de Medicine. 3. Accounts of the proceedings of the different medical focieties in France and of the National Inftitute, as far as they relate to medical fcience.—In the *Introduction,* the editors prefent the principles on which their plan is founded, and which feems likely to meet with approbation. In the two numbers which we have before us, we find, 1. An obfervation on a hydrops cyfticus of the liver by Corvissart and Le Roux. 2. Refearches and obfervations on the fame diforder, by Lassus. 3. Obfervations on an aneurifm of the heart. 4. Obfervations on an anomalous tumour at the fore arm by Cit. Boyer. 5. An hiftorical memoir on the cow-pock, by Cit. Aubert. Two hiftorical memoirs on the medical fchool at Paris. An obfervation on excrefcences placed at the orifice of the aorta; on the fcirrhofity of the heart, &c. by Corvissart and Le Roux. Obfervations on a new a new proceeding of Cit. Boyer in the conſtrictions of the œſophagus, by introducing elaſtic probes, by Cit. Vareliaud. Meteorological obſervations made at Paris in the year 8. Medical conſtitution of Paris during three months of the ſame year. Literary news, extracts of books, &c. conclude each number.

---

*Recherches et Decouvertes ſur la Nature du Fluide nervoux;* i. e. Reſearches and Diſcoveries on the Nature of the nervous Fluid, or vital ſpirit, and on the manner of action, after new and exact experiments by Profeſſor W. Le Febure. Paris, Koenig; and Frankfort, Fr. Eſlinger; year 9.

The author diſcuſſes in the firſt paragraphs ſome general notions, and relates in the ſeventh ſection, in a few words, the ideas moſt generally adopted on the nervous fluid, into the nature of which he enquires in the following paragraphs. He deſcribes the pneumato chemical apparatus, which he uſed for making different curious experiments on the nervous fluid, the reſults of which are as follow. 1. That inflammable air exiſts in the brain and medulla oblongata, as well as in the nerves themſelves and in the ſperma, the moſt elaborate ſecretion of organiſm, where it is mixed with the lymphatic humours, which ſerve it as a vehicle, and with a portion of carbonic acid. 2. That theſe two gaſſes are found in different animals. 3. That they are likewiſe obſerved in the medulla, nervous, and ſeminal parts of females. 4. That the inflammable air has a different ſpecific weight in the different animals, on which account its conſtituent principles muſt be different. 5. The inflammable air which circulates in the nerves, is altered in the morbid ſtate of the body. After having thus ſtated theſe corollaries, the author proceeds to diſcuſs the following queſtions. 1. The nervous fluid conſiſts of inflammable air; but how does this gas circulate in the nervous tubes? This cannot proceed in the ſame manner as the blood circulates in the arteries and veins. How does this air give impulſe to the animal body? How does it vivify it? What is its action and its effect? 2. The nervous fluid is differently combined or modified in different animals; what is the nature of it? 3. The nervous fluid is altered in the diſeaſed ſtate, but in what manner? By what means does nature re-eſtabliſh it to the natural ſtate? Such are the contents of this pamphlet; but whether we ſhall penetrate in this manner the abſtruſe doctrine of the nervous principle, is a matter of farther enquiry, and, we fear, not quite exhauſted by the author of this publication.

---

*Taſchenbuch fur die Geſundheit*; i. e. Pocket Book for Health, for the year 1801; edited by Frederic Hildebrand, Profeſſor at Erlangen, 250 pp. 12mo. Erlanger for Walther.

It is indeed a very uſeful undertaking to communicate medical notices and truths to the public at large; but it requires great care and precaution in chooſing proper materials, and in propoſing them in a proper manner. Mr. Hildebrand, whoſe merits in medical

ſcience are generally acknowledged, affords in the preſent publication both inſtruction and amuſement to his readers, as he unites a proper choice of materials with a pleaſant manner of treating them, ſo that this popular work may be conſidered as one of the beſt of the kind. The author premiſes ſome general rules for the preſervation of health, in which he communicates ſome remarks on what ſick people ought to obſerve, &c. *Firſt ſection,* Rules concerning the different actions to which men are expoſed. Ch. i. of the air, in which he diſcourſes on the conſtituent parts of the atmoſphere, their different actions, the pernicious effects of irreſpirable vapours, &c. and at laſt he treats of the eudiometers. Ch. ii. on the hygrometer, and on the advantages of a dry and warm dwelling. Ch. iii. warmth and cold, on the effects of both, on ſtoves, beds, on freezing to death. Ch. iv. on eating and drinking, whether the food and drink ought to be cold or warm; this depends on cuſtom, on the impurities of the primæ viæ; on food eaſily and difficultly digeſtible; animal food, vegetable food; on ſugar, milk: *liquors* and *drinks*; water, beer, wine, coffee, tea, &c. — on fruits, vinegar, ſpices, ſnuff, tobacco. Ch. v. on the excretions, perſpiration, excretio alvi, urinæ, ſeminis, &c. Ch. vi. on dreſs. Ch. vii. on the geſture and ſituation of the body. Ch. viii. on ſleep. Ch. ix. on motion and reſt. Ch. x. actions of the mind. *Second Section,* Rules with reſpect to the different parts of the body; for the eyes, teeth, breaſt, belly, and ſkin. The contents of this book are inſtructive and pleaſant.

---

*The Family Physician; or Domestic Medical Friend: containing plain and practical Instructions for the Prevention and Cure of Diseases, according to the newest Improvements and Discoveries; with a Series of Chapters on collateral Subjects, comprising every thing relative to the Theory and Principles of the Medical Art, necessary to be known by the private Practitioner. The whole adapted to the Use of those Heads of Families who have not had a classical or medical Education. By* ALEXANDER THOMSON, M. D. Author of a Treatiſe on Nervous Diſorders; of Dialogues in a Library; and other Productions. 12mo. pp. 580, price 6s. in boards. London, 1801. Phillips.

THE work here offered to the public, the author obſerves in his preface, "is ſo intimately connected with human happineſs, that little need be ſaid in favour of its general utility. To preſerve the health of the body, and to cure its diſeaſes, have ever been regarded as objects of great importance to mankind; and a knowledge of the means for promoting theſe ſalutary purpoſes can never be too widely diffuſed. The art, however, of preſerving health is, in general, ſo little cultivated, as well as imperfectly underſtood, that more diſeaſes proceed from a violation of its precepts than from all other cauſes whatever; and, with regard to the cure of them, an early obſervance of their approach, and a prompt application of medicine, are circumſtances which, if unfortunately neglected, neglected, no fubfequent fkill or exertion may ever afterwards be able to retrieve.

"Another circumftance alfo contributes greatly to favour the progrefs of difeafes. People often, from various motives, are difinclined to call for the affiftance of a phyfician, until the diforder has fo far advanced, that neither the diftrefs of the patient, nor the apprehenfions of his friends, can admit of any longer procraftination. Whether the difeafe be chronic or acute, this difeafe proves equally pernicious. If chronic, the difeafe may become fo fixed in the habit as to refift the utmoft efforts of medicine; and if acute, the rapidity of its progrefs may not only bid defiance to all reftraint, but utterly preclude every reafonable hope of recovery.

"Nothing, therefore, can fo effectually obviate thefe inconveniencies as a work of the prefent kind, which not only teaches to difcover a difeafe at an early period, but to apply the proper means, as well for preventing its increafe as, if poffible, for its total extinction.

"The author's principal care, he obferves, has been to defcribe the various difeafes with accuracy, and to recommend fuch a method of cure as is conformable to the lateft eftablifhed improvements in medical practice. In executing this plan, he has every-where endeavoured to be fparing in the ufe of technical expreffions; but the total exclufion of them being incompatible with precifion of fentiment, an explanation of all fuch terms is given in a Gloffary."

In our perufal of this comprehenfive work, we have been particularly ftruck with the concifenefs and perfpicuity with which the fymptoms of the various difeafes are defcribed; and the care every where taken to point out thofe marks or fymptoms which diftinguifh one difeafe from another. This is indeed an effential requifite in a popular work of this kind; for if the difeafe be miftaken, the well intended means of relief may prove fatally pernicious.

In delivering the method of *cure*, the author has very properly accommodated his directions to the underftandings of plain, unlettered parents, and guarded them againft the confequences of ufing deleterious remedies without medical affiftance; we think, however, that even the experienced practitioner will find many valuable hints and improvements not to be found in any other work.

Befides the fubjects ufually treated of in works on the fubject of domeftic medicine, Dr. T. has given the method of treating accidents; an account of the medicinal baths and fprings in this ifland with their ufes; an account of the fubftances moft commonly employed in medicine, more efpecially thofe found in Great Britain, with their virtues and ufes, together with rules for collecting and preparing them. There is alfo, what no good book fhould be without, a proper Index.

Upon the whole, if extenfive refearch, important obfervation, and practical utility, can ftamp a value on a popular medical production, we believe the prefent volume has fuch juft pretenfions to the approbation and favour of the public as cannot fail to enfure its fuccefs.

*A Practical Treatise on Diet, and on the most salutary and agreeable Means of supporting Life and Health, by Aliment and Regimen; adapted to the various Circumstances of Age, Constitution, and Climate; and including the Application of modern Chemistry to the Culinary Preparation of Food. By* WILLIAM NISBET, M. D. Fellow of the Royal College of Surgeons, Edinburgh, Author of the Clinical Guide, &c. 12mo. pp. 430. price 6s. boards. London, 1801, Phillips, &c.

WE cannot introduce the account of this work better than by an extract from the preface and introduction.

"The subject of diet is of the first importance to mankind. It has received, however, from the generality of physicians, a less share of attention than its importance demands, and the cure of actual disease is commonly more aimed at than its prevention, or the real preservation of health.

"Compared with the other branches of the medical art, the works upon diet are few, and of these a very small number can be considered as possessing practical utility. Hence they are little fitted to instruct society at large. Those which do afford practical instruction, consist generally of small detached works on particular subjects; and those which assume a more complete form, are entirely of a professional and scientific nature, and abound in hypothetical and conjectural matter.

"Among the ancient physicians, diet was considered as a subject of the first consequence. Their remedies were fewer in number than those of the moderns, and they were consequently led to the regulation of diet, as a more succesful means of curing disease than the application of medicine. It would have been well for society at present, if the same opinion and practice were followed by modern physicians.

"Of the advantages attending a proper regulation of diet, most individuals can form a judgment from their own experience. It is at least this idea upon which the common maxim is founded, that every man, after the age of forty, is best fitted to be his own physician; a maxim which implies, in other words, that every man, in respect to what he eats and drinks, is enabled to distinguish, by that period, the food which best agrees with him.

"In chronic diseases, it is obvious that the chief means of cure consist in the proper regulation of diet alone. The origin of diseases, it must be allowed, is more frequently to be traced to improprieties in diet, than to any other cause; and the mode of relief must necessarily be sought for in the reversing of that plan of living which gave rise to the diseased state.

"In the following work it has been the leading object of the author to unite the knowledge of particular facts with general principles and reasonings; and to carry these reasonings no further than to connect facts and principles with practice.

"He has collected together a greater mass of matter than has appeared before in any single book, and, without assuming any merit merit to himfelf, his work muft be ufeful entirely on that account. It might, indeed, have been extended to a much larger fize; but his chief labour was to condenfe his materials, that he might enable readers of all defcriptions to acquaint themfelves with a fubject of fuch particular importance, and exhibit every fact, at the fame time, in the fimpleft and cleareft point of view.

"Nothing, indeed, could tend fo much to improve the fcience of medicine, as to endeavour to make man acquainted with himfelf. The principles of the animal œconomy rendered familiar and plain, and a knowledge conveyed of the action of fubftances upon it, is the only way to root out thofe falfe maxims and prejudices which ignorance and education produce.

"How far the prefent attempt has fucceeded, muft be left to the decifion of the public. The endeavours of the author, he can at leaft affert, have been well intended for the benefit of mankind.

"When we confider that in the catalogue of difeafes, at leaft two-thirds are of a chronic nature, or the effect of our own irregularities; the importance of this part ftands in a confpicuous view. By the very inftinct of felf-prefervation, we are more immediately excited to its inveftigation; and by a knowledge of it, feafonably applied, we fhall often have it in our power to prevent difeafe—Even where difeafe has actually occurred, we fhall be enabled by this knowledge to check its progrefs, and at the fame time to affift the efforts of medicine. But while the principles of the animal œconomy, being once rendered familiar and plain, is the only way to root out thofe falfe maxims and prejudices which education and ignorance introduce, there are certain limits beyond which this knowledge fhould not be carried. An acquaintance indeed with the fubject of diet, cannot fail to be attended with the beft confequences; but when this familiar or domeftic kind of knowledge is extended to what is ftyled ftrictly the province of medicine, its influence there is often of the moft fatal tendency,—Medicine is a fcience complicated in its principles, and from the varying appearance of difeafe frequently uncertain. The man, therefore, who, confiding in this fuperficial knowledge, attempts to be his own phyfician, feldom is fo to much purpofe. He is liable to miftakes at every turn he takes, and the mifchief is often irreparable before he is aware of the danger. Even the phyfician who prefcribes for himfelf is not unfrequently led into an error; and the remark made to the friend of one, on an occafion of this kind, "He has a chance of getting well, for he no longer prefcribes for himfelf," contains much truth and juft obfervation.

"At the fame time, while a fuperficial acquaintance with the fcience is thus condemned, we are equally averfe that any myftery fhould hang over it, or that the veil which has been withdrawn from the other branches of knowledge fhould continue its obfcurity here. Let the principles of medicine be once fairly known, and let its precepts be directed by judgment and experience; it will be then of little confequence whether its aid is beftowed by a profeffional hand, or by the zeal of humanity and friendfhip.

To aſſiſt in doing this is the object of the following Treatiſe, in which we ſhall conſider as the extent of our ſubject the various means of ſupporting life, as applied—

1ſt. To the ſurface or external part of the body.

2dly. To the lungs, or what we may term the intermediate ſurface; and,

3dly, To the internal parts, through the proper organ of the ſtomach.

We ſhall next examine the action of the body, as modifying the power of theſe means of ſupport when introduced into the ſyſtem; then conſider the influence of the mind as affecting the body in the ſame way; after which we are naturally led to trace the paſſage of the various alimentary matters from the body, in their different altered and aſſimilated ſtates, through the ſeveral evacuations; and laſtly, we ſhall be prepared to concentrate in one detail the various means for the preſervation of health, and to mark the circumſtances to which the prolongation of life is chiefly to be attributed; concluding this ſyſtematic view of the ſubject with an abſtract of the general principles of chemiſtry, as applied to this part of medicine, firſt in the detection of the component parts of diet, and ſecondly in their various preparation for the purpoſes of nouriſhment and the delicacy of the table.

Thus we ſhall be led to trace the human body as a wonderful ſyſtem of parts:

By the lungs drawing life and heat from the ſurrounding atmoſphere:

By means of the ſtomach ſupplying itſelf with nouriſhment from the various parts of creation, for the preſervation of its animalization and form:

Then removing, firſt, the uſeleſs part of this nouriſhment by the inteſtines:

Secondly, diſcharging the accumulation of the animal principle derived from the ſame nouriſhment by the lungs: and,

Thirdly, giving outlet to the various ſaline products ariſing from the operations of the œconomy, by the kidneys, the ſkin, and the other leſſer excretions."

We are convinced that this compendious work, on the important ſubject of Diet, will prove a valuable and acceptable preſent to the public.

---

*Practical Obſervations on the Nature and Treatment of ſome exaſperated Symptoms attending the Venereal Diſeaſe.* By EDWARD GEOGHEGAN, Member of the Royal College of Surgeons; of the Royal Medical Society, Edinburgh; and Surgeon to the Dublin General Diſpenſary. 12mo. pp. 75, price 3s. London, 1801, Debrett, &c.

"I propoſe," ſays the author in his introduction, "to conſider ſome ſymptoms attending the venereal diſeaſe, the nature and treatment of which, I think, are not well underſtood; and I am the more more defirous of entering into the inveftigation, finding that the moft refpectable modern authorities are not only undecided in their opinions, but inculcate a practice, which appears to me highly injudicious, and from which I have witneffed the moft deftructive confequences. The variety of forms which this difeafe affumes, and the fymptoms of extraordinary malignity which occafionally occur, invole its treatment in confiderable difficulty: there is fcarcely a difeafe which it does not refemble in fome of its features, and there is no general plan of treatment which is not contraindicated, under fome particular circumftances; hence in inveftigating its phenomena, an extenfive field of refearch prefents itfelf, and indeed we have to lament that in confidering the varieties in this difeafe, profeffional men calculate by fo very limited a fcale.

" Although almoft every form which the venereal difeafe prefents, furnifhes ample matter for obfervation, I fhall confine myfelf to fome aggravated fymptoms, in which I have hadconfiderable experience.

" The fymptoms termed phymofis is that to which I particularly allude; and I know of no affection, the event of which is more interefting; it frequently terminating in mortification, and the lofs of a part or of the entire of the penis. The number of inftances of this kind, which occurred during the fummer, autumn, and winter of 1799, excited my aftonifhment, and on communicating with other practitioners, I found that they had met with fimilar cafes in a much greater number than at any former period: as to the nature and treatment of it, there were a variety of opinions, in general different from thofe I had formed.

" It was noticed by the public, that the venereal difeafe then raging appeared to be fingularly malignant; and I have heard even profeffional men fay, that they thought there was an increafed degree of virulence in the infection. The appearances which gave rife to thefe remarks, were violent tumefactions of the penis often terminating in gangrene, particularly when injudicioufly treated; other fymptoms were alfo obfervable, fingular for intenfity of degree. Although I was always decided in the opinion and practice I now maintain, yet the frequent inftances within fo limited a time, afforded opportunities for much obfervation, and led me to queftion the propriety of the practice generally purfued and recommended.

" When the ordinary fymptoms attending an infectious difeafe appear to be exafperated in an unufual degree, the queftion arifes, to what are we to attribute this increafed degree; whether to increafed acrimony in the poifon, or to any adventitious or phyfical caufes infenfibly operating? This is the pivot upon which the point of practice muft turn. If to the former, mercury is the remedy; if to the latter, a great variety of circumftances are to be taken into confideration, which are too frequently neglected. There is nothing fo common as to hear the furgeon declare in every cafe which does not yield to the ufual mercurial difcipline, that the conftitution is in fault, and his mode of rectifying it is, in general, the the free uſe of bark and wine, opium, cicuta, decoctions of the woods, ſea-bathing, and thoſe means ſometimes are conjoined, but generally follow mercury in a kind of routine, as if they had a ſpecific operation in every diſeaſe connected with the venereal, or with its antidote. Before I proceed to enquire into thoſe cauſes, which, I conceive, are but little attended to in accounting for the varieties in this diſeaſe, I think it neceſſary to notice the opinions of Mr. John Hunter on this part of the ſubject; he ſays; 'That when this tumefaction takes place, in conſequence of a chancre, he ſuſpects there is an irritable diſpoſition in the habit, for it is plain there is more than the ſpecific action; the inflammation extending beyond the ſpecific diſtance.' In his directions for the conſtitutional treatment he ſeems a good deal puzzled — his words are, 'In thoſe caſes, where violent inflammation has attacked the ſeat of a chancre producing phymoſis as before deſcribed, and often ſo as to threaten mortification, a queſtion naturally ariſes — Is mercury to be given freely to get rid of the firſt cauſe? Nothing but experience can determine this; I ſhould incline to believe, that it is neceſſary that mercury ſhould be given, for I am afraid our powers to correct ſuch a conſtitution, whilſt the firſt cauſe ſubſiſts, are too weak; however, on the other hand, I believe the mercury ſhould be given ſparingly, for if it aſſiſts in diſpoſing the conſtitution to ſuch ſymptoms, we are gaining nothing, but may loſe by its uſe; I therefore do ſuppoſe, that ſuch medicines as may be thought neceſſary for the conſtitution, ſhould be given liberally: as well as the ſpecific, bark is the medicine that probably will be of moſt general uſe; opium, in moſt caſes of this kind will alſo be of ſingular ſervice; the bark ſhould be given in large quantities, and along with it mercury, whilſt the virus is ſtill ſuppoſed to exiſt; or if the inflammation has ariſen early in the diſeaſe, they may then be given together, ſo as to counteract both diſeaſes, and not to allow the inflammation to come to ſo great an height as it would otherwiſe do, if mercury was given at firſt alone. This inflammation may be ſo great in many caſes, or be ſo predominant, that mercury may increaſe the diſpoſition, and therefore become hurtful. Where this may be ſuppoſed to be the caſe, bark muſt be given alone.'

"Theſe are his obſervations in full, on the conſtitutional treatment of phymoſis; what is to be learnt from them, I am at a loſs to diſcover; he puts a caſe of inflammation threatening mortification; after expreſſing many doubts he adviſes mercury, but that it ſhould be given ſparingly leſt it ſhould do *harm*; in the next lines he recommends it accompanied with bark and opium, and concludes by telling us, it may become *hurtful* in the very caſe in which he adviſes it. It is evident from theſe equivocal and inconſiſtent opinions, that he had not come to any fixed or determined principle as to the nature of the diſeaſe, or mode of treatment; the ſurgeon who gives mercury, and he who does not, in this threatened mortification, are alike ſanctioned by his authority; he alſo adviſes in the local treatment, to inject mercurials, even corroſive rosive sublimate in the proportion of one grain to an ounce of water, and other mercurials to remain in contact with the parts, but concludes, that he has his doubts as to the propriety of using any irritating applications in such cases.

" With respect to the question, whether the increased acrimony of the poison has any share in producing those aggravated symptoms? here, it is necessary to take a view of the effects which usually attend its application in the first instance. When applied to a non-secreting surface, ulceration is generally the consequence, and although attended with some degree of inflammation, yet it is rather circumscribed, and the ulcerative process goes on more rapidly than the inflammatory, and the latter is often totally wanting. Females having the slightest appearances, without even ulceration or any inflammatory symptom; indeed, ignorant of being infected, constantly communicate the disease, and the persons receiving it are variously affected; in one man it will exhibit the most trivial, in another the most dreadful appearances, and both infected by the same woman, at nearly the same time; taken into the stomach it produces no effect, and even proves harmless when applied to the surface of many persons; it also remains in the constitution for years, without manifesting itself, or exciting the least disturbance. In the small-pox, we every day see the mildest and most malignant kind, and both produced from the same infection. These facts establish the principle most unequivocally, that mild or violent symptoms, whether attended with inflammation or ulceration, or in whatever form they appear, are not characteristic of variety in the matter of infection; hence, we cannot account for aggraved symptoms, from the nature of the poison. We are led then to look for an explanation of the phœnomenon (peculiarity of constitution is the generally admitted cause) to some other cause, and whilst I agree that it is the true source, I cannot but express my astonishment at the narrow view which is generally taken of this material point. One would think from the plans of cure laid down and usually pursued, that bad constitution meant some fixed and definite thing, for which there was a decided rule of treatment; not that fluctuating state of the animal machine which is liable to vary with every breeze.

" Surely, in considering the constitution, the great variety of circumstances which influence it are are to be taken into the account. The constitution of the air, place of residence, disposing to diseases of different types, disposition to particular diseases, effects of the human passions, intemperance, exercise, where rest is necessary, habits of life, also neglect of the local sore, or general habit, and many other causes of interrupting the general health, which it is impossible to recount, and all of which have their share in exasperating diseases, and changing their form. Many states of constitution may arise during the treatment of the different stages of the venereal disease, from some of the causes enumerated, in which mercury would be contraindicated, it is easy to conceive, that inflammations of the penis may be superinduced, whether chancre cre exifts or not, as in gonorrhœa; and that chancres may fpread and put on the moft malignant appearances, independant of the virus. When the penis becomes the feat of difeafe, its irritability is preternaturally increafed. Should any additional caufe of difeafe operate locally or generally, it is obvious that the part in a ftate of morbid fenfibility, will feel its effects in a greater degree than any other, and that new fymptoms will be produced *quo ad injuriam*. Was a perfon with a chancre to receive a hurt on his penis, and violent fymptoms to enfue, could any thing be more abfurd than to treat the cafe as venereal during the new fymptoms? And might not the fame effects arife from an injury of the conftitution? Do we not every day fee the moft violent difeafes come on fuddenly from an acceffion of cold, and affecting thofe parts particularly which were previoufly in a morbid ftate? Thofe who are fubject to difeafes of the urinary organs, gout, rheumatifm, ophthalmia, fore throat, &c. &c. expect a vifit from their old complaints, at thofe feafons when the weather is remarkably variable, as in fpring and autumn. Hippocrates obferves, "*Mutationes temporum maximè pariunt morbos, et in ipfis temporibus, magnæ mutationes aut frigoris aut æftus aliaque congruentur rationi eodem modo.*"

"At thofe periods when catarrh is a frequent difeafe, and which is generally occafioned by an epidemic conftitution of the air, it is obferved, that pre-exifting difeafes are increafed, and that the prevailing epidemic manifefts itfelf in a variety of forms. Sydenham obferves, that 'At the time of an epidemic, every other diforder, in fome meafure, participates of the nature of the reigning epidemic.' All the accounts we have of difeafes arifing from the ftate of the atmofphere, give inftances of the variety of forms in which they appear, although the prevailing difeafe was catarrh, eryfipelas often terminating in gangrene and death, palfies, convulfions, fudden deaths, mania, &c.

"That the principle contended for is admitted by every medical philofopher, there can be no queftion; and, I prefume, that its application in explaining the phenomena under confideration is obvious. I have already endeavoured to explain, how difeafes in the penis may be aggravated; and the circumftances of the aggravated fymptoms occurring when the fores are nearly healed, and the fyftem fully under the influence of mercury, prove, that they are not caufed by venereal irritation, and that the virus has no other fhare in the difeafe, only in as much as it predifpofes the part to be acted on by the remote caufes; a morbid condition of body alfo is induced by mercury, which renders it peculiarly liable to adventitious difeafes. Thefe obfervations apply to every fymptom attending the venereal difeafe, all which may be aggravated from fimilar caufes."

We are convinced, as no doubt our readers are, that this Writer's *Obfervations* merit the ferious attention of practitioners.

MEDICAL

